# Utility of Direct Immunofluorescence in Cutaneous Autoimmune Bullous Disorders

**DOI:** 10.7759/cureus.14562

**Published:** 2021-04-19

**Authors:** Arika Brar, Abhimanyu Sharma, Samal Nauhria, Shreya Nauhria, Aniruddha Bhattacharjee, Jagannadha Peela, Kusum Joshi

**Affiliations:** 1 Department of Pathology, Swai Man Singh Medical College and Hospital, Jaipur, IND; 2 Department of Pathology, Maharishi Markandeshwar University, Ambala, IND; 3 Department of Pathology, St. Matthew's University, Georgetown, CYM; 4 Department of Psychology, University of Leicester, Leicester, GBR; 5 Department of Physiology, St. Matthew's University, Georgetown, CYM; 6 Department of Biochemistry and Genetics, St. Matthew's University, Georgetown, CYM

**Keywords:** direct immunofluorescence, autoimmune bullous disorders, bullous dermatoses, histopathology (hp)

## Abstract

Background

Autoimmune bullous disorders (AIBD) are a heterogeneous group of disorders with substantial clinical overlap associated with blistering of skin or mucosa.

Aims

The present study aimed to study the histopathological spectrum and evaluate the utility of direct immunofluorescence (DIF) on snap-frozen and paraffin-embedded sections in resolving the differential diagnosis of AIBD and connective tissue disorders of the skin. We also compared the efficacy of DIF on paraffin versus the snap-frozen sections in diagnosing AIBD.

Methods

The present study was conducted for three years (2017-2019) and included 27 biopsies. We also included a retrospective analysis that included 25 biopsies collected over three years (2014-2017). Histopathological examination and DIF were conducted on all samples.

Results

Pemphigus vulgaris was the most common autoimmune cutaneous disorder constituting 37% (n = 10) in prospective and 36% (n = 9) in the retrospective study. DIF showed a specificity of 81.25% in our prospective study. While on the paraffin-embedded sections, it showed a specificity of 66.6% in our retrospective study. In the prospective study, DIF on paraffin-embedded sections had a positivity rate of 43.75% as compared to 81.25% in DIF done on snap-frozen sections.

Conclusion

DIF is a sensitive tool for the diagnosis as well as distinguishing immune-mediated bullous disorders from other lesions primarily when performed on snap-frozen sections. The diagnostic yield is enhanced by DIF in cases that pose a diagnostic dilemma both clinically and histologically. The final diagnosis depends on all clinical, histopathological and immunofluorescence findings.

## Introduction

Autoimmunity plays a significant role in the etiology of various skin disorders [1,2]. The clinicopathologic spectrum of autoimmune bullous disorders (AIBD) is heterogeneous; thus, accurate diagnosis becomes essential for effective treatment and prognosis. Difficulties in diagnosis arise as the lesions closely mimic each other, and the evolution of lesion morphology with therapy [2,3].

In practice, light microscopic findings, combined with direct immunofluorescence (DIF) results, are used for a definitive pathological diagnosis [4,5].

DIF microscopy is considered the gold standard for differentiating various vesiculobullous diseases [6,7]. In addition to diagnosis, DIF also aids in monitoring response to therapy and predicting relapse [3,5].

The present study aimed to study the histopathological spectrum and evaluate the utility of DIF on snap-frozen and paraffin-embedded sections in resolving the differential diagnosis of AIBD and connective tissue disorders of the skin. We also compared the efficacy of DIF on paraffin versus the snap-frozen sections in diagnosing AIBD.

## Materials and methods

This study was conducted over three years (2017-2019) in the Department of Pathology at Maharishi Markandeshwar Institute of Medical Sciences and Research (MMIMSR). All cases with a suspected clinical diagnosis of cutaneous autoimmune disorder were included. Study approval was taken from the MMIMSR review board and institutional ethics committee (IEC/MMIMSR/16/195) and consent was taken from the patients. Along with prospective cases, we also included three years (2014-2017) of available retrospective case data. For prospective cases, two biopsies were processed; one in Michel transport medium for DIF and the other in 10% buffered formalin for routine paraffin processing. Biopsies for DIF were embedded in optimal cutting temperature (OCT) followed by cold acetone dips and sectioned at 4-µm thickness in a cryostat. Similarly, the paraffin-embedded sections were deparaffinized and rinsed with Tris buffer before treatment with Proteinase K. Sections so processed, were then incubated with fluorescein isothiocyanate-conjugated antisera directed against human IgG, IgM, IgA, and C3 and finally viewed under Nikon Epifluorescence- Eclipse microscope.

DIF results were recorded by considering the nature, location, extent, and pattern of immune deposits. All the formalin-fixed biopsies were routinely processed and stained with hematoxylin and eosin (H&E). These slides were reviewed for the presence of the lesions and correlated with DIF findings. For the retrospective cases, we included histopathologic examination (HPE) of paraffin-embedded H&E sections and DIF on paraffin sections. The final diagnosis was based on a combination of the suspected clinical diagnosis along with HPE and DIF findings.

## Results

The diagnostic spectrum of all the included cases is shown in (Table 1).

**Table 1:** Diagnostic spectrum of all the cases (n = 52).

Diagnostic spectrum	Cases (n = 52)		Overall percentage (%)
	Prospective (n = 27)	Retrospective (n = 25)	
Pemphigus vulgaris (PV)	10	9	36.5
Bullous pemphigoid (BP)	3	6	17.3
Dermatitis herpetiformis (DH)	1	1	3.8
Chronic bullous dermatoses of childhood (CBDC)	1	0	1.9
Sub-corneal pustular dermatoses (SCPD)	1	0	1.9
Prurigo nodularis (PN)	1	0	1.9
Lichen planus (LP)	3	1	7.7
Leucocytoclastic vasculitis (LCV)	3	0	5.8
Paraneoplastic pemphigus (PP)	0	1	1.9
Pemphigus foliaceous (PF)	0	1	1.9
Epidermolysis bullosa (EB)	0	1	1.9
Darier's disease	0	1	1.9
Epidermolysis bullosa aquisita (EBA)	0	1	1.9
Discoid lupus erythematous (DLE)	0	3	5.8
Descriptive	4	0	7.7
Total	27	25	

Prospective cases

There were 27 prospective cases out of which 16 patients were of AIBD. Amongst the 16 cases, PV constituted the most common AIBD constituting 62.5% (10 out of 16), followed by BP constituting 18.8% (three out of 16) cases. The most commonly affected age group was 31-40 (37.5%), followed by the age group of >50 (18.5%) and 21-30 (12.5%) years. Males and females were equally affected in the age group of 31-40 years. The mean age of the studied population was 38.6 years. Out of 16 patients, nine were males, and seven were females.

The distribution of the plane of separation is shown in Table 2.

**Table 2:** Distribution of plane of separation in prospective cases DEJ: dermo-epidermal junction; HPE: histopathologic examination.

Diagnostic spectrum (n = 16)	Plane of separation					No bullae
	HPE not done	Sub-corneal	Intra-epidermal	Supra-basal	DEJ	
Pemphigus vulgaris (n = 10)	1 (10%)	0	0	7 (70%)	0	2(20%)
Bullous pemphigoid (n = 3)	0	0	0	0	3 (100%)	0
Dermatitis herpetiformis (n = 1)	1 (100%)	0	0	0	0	0
Chronic bullous dermatoses of childhood	0	0	1 (100%)	0	0	0
Sub-corneal pustular dermatoses of childhood (n = 1)	0	1 (100%)	0	0	0	0

**Figure 1:**
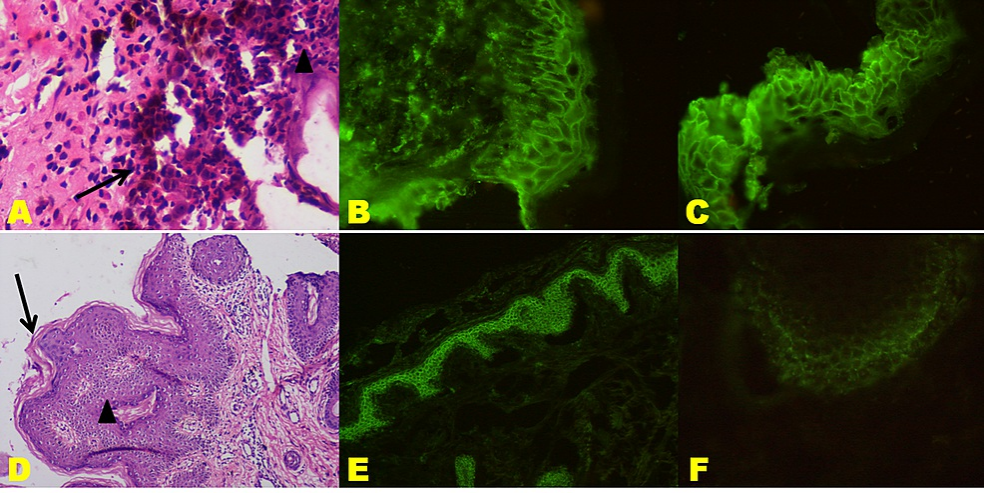
Pemphigus vulgaris; A: Biopsy exhibiting suprabasal cleft containing acantholytic cells (arrow) and few neutrophils (arrowhead) (H&E;400X). B & C: Fishnet pattern deposition of IgG and C3, respectively, in squamous ICS (DIF;400X). D: Biopsy from perilesional skin exhibiting epidermal papillomatosis (arrow) and mild acanthosis (arrowhead) with no bullae (H&E;100X). E & F: Fishnet pattern deposition of IgG and C3, respectively, in perilesional ICS (E-DIF;100X; F-DIF;400X). DIF: direct immunofluorescence; H&E: hematoxylin and eosin.

Out of the total 10 PV cases, the sample was not available for one case. For the remaining nine HPE was carried out, and two cases showed no bullae. Out of these two, there was one case with perilesional biopsy, and the other was a known case of PV under treatment. The content of bullae predominantly showed acantholytic cells (seven cases) and neutrophils (five cases), seen in (Figure 1A). Only one case revealed suprabasal bullae containing mixed inflammatory infiltrate consisting of neutrophils and eosinophils. Lymphocytes were also found in the suprabasal bullae of one, while a row of tombstones formed the base of the bullae in four cases.

The three BP cases revealed bullae at the dermo-epidermal junction (DEJ), and bullae content showed neutrophils’ predominance in two cases. Among these two, only one case showed the presence of acantholytic cells along with neutrophils. Mixed inflammatory infiltrates consisting of eosinophils and neutrophils were also seen in one case. HPE for DH was not carried out since the sample was not available. However, DIF was done on the available frozen section confirming the diagnosis of DH. CBDC bullae showed a mixed inflammatory infiltrate and the absence of acantholytic cells, while SCPD bullae had predominant neutrophils with few eosinophils.

DIF was conducted on all the 27 skin biopsies, which were analyzed based on type, site and pattern of antibody deposited. DIF was positive in 13 biopsies which included nine cases of PV, two cases of BP, one case of DH and one case of CBDC. Fourteen patients showed no deposits. The type and site of deposited antibodies are shown in Table 3.

**Table 3:** Type and site of antibody deposition in various AIBD (prospective cases). IgA: immunoglobulin; IgG: immunoglobulin G; IgM: immunoglobulin M; DEJ: dermo-epidermal junction; AIBD: autoimmune bullous disorders.

Diagnostic spectrum (n = 16)	Type of antibody deposition					Site of antibody deposition			
	No deposits	IgG	IgA	IgM	C3	No deposits	ICS	DEJ	Dermal
Pemphigus vulgaris (10)	1 (10%)	9	0	0	5 (50%)	1	9	0	0
Bullous pemphigoid (3)	1 (33.3%)	0	0	0	2 (66.7%)	1	0	2	0
Dermatitis herpetiformis (1)	0	0	1 (100%)	0	0	0	0	0	1
Chronic bullous dermatoses of childhood (1)	0	0	0	0	1 (100%)	0	0	1	0
Sub-corneal pustular dermatoses (1)	1 (100%)	0	0	0	0	1	0	0	0

Fish-net or lace-like pattern of antibody deposition at the ICS was noted in all nine cases of PV, which were positive on DIF. One case of PV showed both fish-net as well as linear IgG at ICS and DEJ, respectively (Figure 1B, 1C). Granular deposition of antibodies at the tip of dermal papillae was seen only in the case of DH. Linear deposition of antibodies was seen in the cases of BP and CBDC. Immunofluorescence on the paraffin section was conducted on all the 27 biopsies, out of which seven cases (six PV; one BP) showed antibody deposition. (Figure 1D-1F). The utility of DIF in the diagnosis of AIBD is shown in Table 4.

**Table 4:** Utility of DIF in the diagnosis of AIBD. Contributary: DIF correlated and thus confirmed the HPE diagnosis. Essential: DIF helped in final diagnosis whereas HPE could not. Non-contributory: DIF could not help with diagnosis and HPE was the final diagnosis. DIF: direct immunofluorescence; AIBD: autoimmune bullous disorders; HPE: histopathologic examination.

Diagnostic spectrum (n = 16)	DIF was contributory	DIF was essential	DIF was non-contributory
Pemphigus vulgaris (n = 10)	8 (80%)	1 (10%)	1 (10%)
Bullous pemphigoid (n = 3)	2 (66.6%)	0	1 (33.4%)
Dermatitis herpetiformis (n = 1)	0	1 (100%)	0
Chronic bullous dermatoses of childhood (n = 1)	0	1 (100%)	0
Sub-corneal pustular dermatoses (n = 1)	0	0	1 (100%)

A comparison of DIF on the frozen section and paraffin section in prospective cases is shown in Table 5.

**Table 5:** Comparison of DIF on frozen section and paraffin section in AIBD (prospective cases). DIF: direct immunofluorescence; AIBD: autoimmune bullous disorders.

Diagnostic spectrum (n = 16)	Diagnosis on DIF of frozen section		Diagnosis on DIF of paraffin section		Sensitivity of DIF on frozen section	Sensitivity of DIF on paraffin section
	Positive	Negative	Positive	Negative		
Pemphigus vulgaris (n = 10)	9	1	6	4	90%	60%
Bullous pemphigoid (n = 3)	2	1	1	2	66.60%	33.40%
Dermatitis herpetiformis (n = 1)	1	0	0	NA	100%	NA
Chronic bullous dermatoses of childhood (n = 1)	1	0	0	1	100%	0
Sub-corneal pustular dermatoses of childhood (n = 1)	0	1	0	1	0%	0

Retrospective cases

According to our retrospective study, the most commonly affected age group was over 50 years (40%), followed by the age group of 41-50 years (28%). The mean age of the study population was 46.16 years. Out of 25 patients, 10 were males and 15 females. The distribution of retrospective AIBD cases based on the plane of separation is shown in Table 6.

**Table 6:** Distribution of plane of separation in various AIBD (retrospective cases). AIBD: autoimmune bullous disorders; DEJ: dermo-epidermal junction.

Diagnostic spectrum	Plane of separation			No bullae
	Intra-epidermal	Supra-basal	DEJ	
Pemphigus vulgaris (n = 9)	6 (66.6%)	3 (33.3%)	0	0
Bullous pemphigoid (n = 6)	3 (50%)	0	3 (50%)	0
Dermatitis herpetiformis (n = 1)	0	0	1 (100%)	0
Paraneoplastic pemphigus (n = 1)	1 (100%)	0	0	0
Pemphigus foliaceous (n = 1)	0	0	0	1 (100%)
Epidermolysis bullosa (n = 1)	0	0	1 (100%)	0
Darier's disease (n = 1)	0	0	0	1 (100%)
Epidermolysis bullosa acquisita (n = 1)	0	0	1 (100%)	0

**Figure 2:**
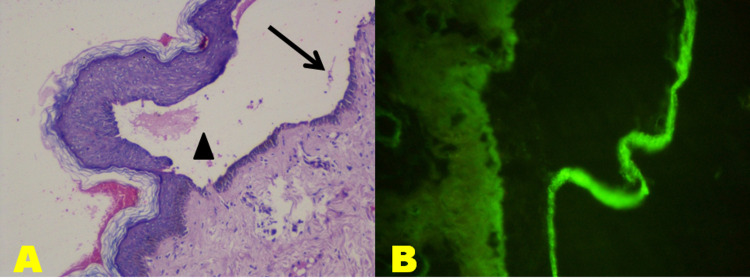
Bullous pemphigoid. A: Photomicrograph of bullous pemphigoid showing intraepidermal bullae (arrowhead) with occasional acantholytic cells (arrow) and paucity of inflammatory cells (H&E; 100X). B: Photomicrograph of bullous pemphigoid showing linear deposition of IgG at the DEJ (IF on paraffin section). H&E: hematoxylin and eosin; IgG: immunoglobulin G; DEJ: dermo-epidermal junction.

Content of bullae in PV predominantly showed acantholytic cells in six and neutrophils in two instances. Six cases revealed bullae with a mixed inflammatory infiltrate comprising neutrophils, eosinophils and lymphocytes, while one showed an inflammatory infiltrate rich in lymphocytes. The row of tombstones formed the base of bullae in four cases. Out of six BP cases, three showed bullae at the DEJ, while the other three showed intraepidermal bullae (Figure 2A). These were initially diagnosed PV on HPE, but after IF on the paraffin section they were confirmed as BP. Bullae content showed a predominance of neutrophils in two cases. Among these two, only one showed the presence of acantholytic cells along with neutrophils. A mixed inflammatory infiltrate consisting of eosinophils and neutrophils was seen in four cases.

DH revealed subepidermal separation with an accumulation of neutrophils and eosinophils at the dermal papillae forming microabscesses. PP revealed the presence of intraepidermal bullae filled with neutrophils. The presence of dermal-epidermal cleft with ballooning was noted. Superficial fungal infection was also seen. Initially, on HPE, diagnosis of this case was given as suggestive of IgA Pemphigus, but on conducting IF on its paraffin section, it was confirmed as PP. EB showed subepidermal bullae with a bullous cavity devoid of any inflammatory infiltrate.

Darier’s disease showed suprabasal cleft containing acantholytic cells. The basal epithelium underneath the cleft showed papillae lined by a single layer of basal cells (villi) projecting into the cleft. EBA revealed only a small part of the epidermis. Hence no definitive diagnosis on the HPE could be made. The diagnosis was confirmed after IF on the paraffin-embedded section and correlating clinically.

IF was conducted on all the 25 paraffin-embedded biopsies. Ten cases showed no deposits. The type and site of deposited antibodies are shown in Table 7.

**Table 7:** Type and site of antibody deposition in various AIBD (retrospective cases). IgG: immunoglobulin G; IgA: immunoglobulin A; DEJ: dermo-epidermal junction; AIBD: autoimmune bullous disorders.

Diagnostic spectrum (n = 21)	Type of antibody deposition				Site of antibody deposition			
	No Deposits	IgG	IgA	C3	No deposits	Around acantholytic cells	DEJ	ICS
Pemphigus vulgaris (n = 9)	3 (33.3%)	6 (66.7%)	0	0	3 (33.3%)	2 (22.2%)	0	4 (44.4%)
Bullous pemphigoid (n = 6)	0	5 (83.3%)	0	1 (16.7%)	0	0	6 (100%)	0
Dermatitis herpetiformis (n = 1)	1 (100%)	0	0	0	1 (100%)	0	0	0
Paraneoplastic pemphigus (n = 1)	0	1 (100%)	0	0	0	0	0	1 (100%)
Pemphigus foliaceous (n = 1)	1 (100%)	0	0	0	1 (100%)	0	0	0
Epidermolysis bullosa (n = 1)	1 (100%)	0	0	0	1 (100%)	0	0	0
Darier’s disease (n = 1)	1 (100%)	0	0	0	1 (100%)	0	0	0
Epidermolysis bullosa acquisita (n = 1)	0	1 (100%)	0	0	0	0	1 (100%)	0

Fish-net or lace-like pattern at the ICS was noted in three cases of PV. Linear deposition was noted in all the six BP, two PV and one case of EBA. Granular deposition of antibody was noted in the pemphigus group only (one PV and PP case each).

Out of 21 cases of AIBD, seven showed no antibody deposition on IF of its paraffin sections. Of the nine PV cases, IF showed a contributory correlation in six with classical fish-net IgG in three, granular IgG in one and linear IgG in two cases. IF was non-contributory in the remaining three cases as there was no antibody deposition. IF was essential in making the diagnosis of three BP cases (50%) reported as PV based on HPE, but with IF conducted on the paraffin section, they showed linear IgG at the DEJ. For the remaining three BP cases (50%), IF was contributory as it showed classical linear IgG at DEJ in two cases and linear C3 at DEJ in one case (Figure 2B).

DIF was also essential in making the diagnosis of one case of Paraneoplastic pemphigus. HPE reported this case as suggestive of IgA Pemphigus, but on conducting the IF on its paraffin section, granular IgG was found at ICS. Similarly, DIF was also essential in confirming EBA as no definitive diagnosis could be made on HPE, but IF on the paraffin section of this case showed linear IgG at DEJ. Thus, the final diagnosis of this case was made after clinical correlation.

## Discussion

The diagnostic value of DIF has been presented by various authors as the lesions closely mimic each other [3,8-11]. The maximum number of cases in the present study were of PV, followed by BP. Contrary to ours, researchers have found the maximum proportion of BP out of all AIBD cases [12,13]. However, our results corroborate the findings of plenty of previous studies [11,14-17]. Various authors have described 100% cases of PV showing suprabasal bullae similar to the current prospective study, in which 70% of PV cases showed suprabasal plane of separation [9,13,16,18]. However, in the retrospective PV cases, 33.3% showed suprabasal bullae, while the remaining 66.6% showed an intraepidermal plane of separation. This was in line with previous literature [19].

The majority (30.9%) of patients were in the age group of 41 to 50 years, which matched those observed in earlier studies [10,11]. In the present study, PV affected middle-aged (mean age 37.2 years), which seems to be consistent with previous research [14]. Contrary to the literature our study found that BP was common in younger individuals (mean age 25 years) [11,13].

In the current study, neutrophils were the predominant inflammatory cells in PV and BP. However, in retrospective cases, eosinophils were the predominant inflammatory cells in BP. Previous studies have shown similar findings [10].

In agreement with previous studies, PV was the most typical lesion in the pemphigus group, with females preponderance [8,9,13,20,21]. Acantholytic cells were seen in 70% cases of PV which is comparable to those observed by various studies [13,16,19]. In line with previous studies, the row of tombstone appearance was seen in 40% of cases [16]. However, some studies have shown a lower percentage [19]. BP results showed a male preponderance, contrary to some studies [22,23].

Subepidermal bullae were seen in 100% cases in the present study, which was similar to the study by Nishioka et al. [24]. However, in retrospective cases, 50% cases of BP showed intraepidermal bullae. These cases were thus diagnosed as PV on HPE but on conducting DIF on the paraffin section of these cases they were confirmed as BP. Inflammatory cells were noted in bulla (100%) and dermal infiltrate (100%) similar to a study [25].

In the prospective study, biopsy for DH was not available for HPE. However, there was one case of DH in the retrospective study showing subepidermal bullae and papillary microabscesses.

In line with some previous studies, a high overall positivity rate of DIF in our prospective study (81.25%) was seen [12]. While some studies have shown a lower positivity rate than our research [15,26]. However, IF on the paraffin sections showed positivity in 14 cases with 66.6% specificity in our retrospective study. The positivity rate of DIF in cases of PV was lower than in some previous studies [9,27].

The treatment status of the negative DIF indicates prolonged remission. DIF had a sensitivity of 66.6% in cases of BP in our prospective study. The findings in our research matched those observed in earlier studies [15,26].

The false negativity on DIF in BP cases can be attributed to the absent epidermis and the longer stay of skin biopsies in transport media (normal saline in our study) and on the site of biopsy. However, a specificity of 100% was observed on DIF on the paraffin section in our retrospective cases of BP. Only one case of DH was received for examination and showed positivity on DIF. DIF proved to be 100% specific in making the diagnosis of this case as there was no biopsy available for HPE. This is in line with previous research [26]. DIF was also 100% specific in confirming the diagnosis of CBDC.

In the current study, most PV cases showed IgG (90%) and C3 (50%) deposition at the ICS. This was in concordance with previous research [28]. IgG and C3 are the major autoantibody and complement, respectively, which are involved in the pathogenesis of PV. IgG showed a higher percentage in our study when compared to some previous studies [10]. However, one known case of pemphigus under treatment showed no antibody deposition indicating its prolonged remission. In our study, 66.7% of BP (two out of three cases) showed DIF positivity with a linear complement at the DEJ. No deposition of any antibody was noted in one case of BP. The previous studies have yielded variable results [11,12].

In accordance with the literature, our study showed classical granular IgA at the dermal papillae tip at DEJ for the single reported DH case [11]. Few studies have shown IgG and IgA deposition, around 50% have demonstrated only IgG deposit, while others have shown no antibody deposition in DH cases [15].

In the present study, nine out of 10 cases (90%) showed classical fish-net IgG deposition at the ICS. As discussed earlier, false negativity in one case of PV was due to the patient’s treatment status. Kabir et al. described (seven out of eight) cases of PV with ICS deposition and (one out of eight) that had granular deposition along the BMZ [12]. The current study described linear complement at DEJ in two out of three cases (66.7%) of BP. However, in some studies, all BP (100%) cases showed linear deposition at DEJ [12,28].

Out of 10 clinically suspected cases of PV, seven (70%) were diagnosed with HPE, while in the other three (30%), HPE was inconclusive. Among these biopsy for HPE was not available for one case, and hence no histopathological conclusion could be made. The remaining two cases showed no bullae on HPE. One of these cases, with no bullae, was the known case of pemphigus on treatment, and for the other case, the perilesional biopsy was sent for HPE. However, conducting DIF on these cases, showed 90% sensitivity as nine out of 10 cases showed the classical fish-net appearance of IgG at the ICS. Only one known case of pemphigus on treatment showed no antibody deposition on DIF. Thus treatment status of this patient resulted in the false negativity of DIF. According to Buch et al, DIF is a very reliable diagnostic test for pemphigus, which becomes positive at an early stage and remains positive for a long period after clinical remission [11].

On the contrary, all three BP cases showed subepidermal bullae at DEJ, giving 100% sensitivity on HPE. However, on DIF, only two cases (66.7%) showed antibody deposition at DEJ, hence proving HPE to be essential and IF only a supplementary test to confirm BP’s diagnosis. The absence of antibody deposition can be due to the longer stay of skin biopsies in transport media or inappropriate biopsy sites. DIF was essential in making the diagnosis for DH as there was no sample available for HPE. Although the case of CBDC showed intraepidermal bullae, DIF proved to be crucial in confirming the diagnosis. It showed a characteristic linear IgA at DEJ thus separating it from DH, where there is granular IgA at DEJ and from BP, where there are linear IgG and C3 along DEJ. However, in a study conducted by Mutasim et al., HPE for the clinically suspected cases of CBDC was inconclusive as there was an absence of bullae on microscopy and were diagnosed only on DIF [29].

In line with our study, Mera et al. compared DIF on formalin-fixed paraffin-embedded with IF of snap-frozen specimens and found that the staining in the sections embedded in paraffin wax was less bright. Researchers also noted that there was a higher chance of negatives in the paraffin sections. However, comparing the results of IF on the paraffin-embedded section with the HPE of the retrospective cases showed comparable results [30].

## Conclusions

From the above discussion, we can conclude that DIF is a sensitive tool for diagnosing AIBD and distinguishing them from other lesions. The diagnostic yield is enhanced by DIF in cases that pose a diagnostic dilemma both clinically and histologically, but the final diagnosis depends on combined clinical, HPE, and DIF findings.
